# Supplementary material for: From sequence to enzyme mechanism using multi-label machine learning
Source: BMC Bioinformatics. 2014 May 19;15:150. doi: 10.1186/1471-2105-15-150 (PMC4229970; doi:10.1186/1471-2105-15-150)
Supplement: Additional file 2 — Java code of ml2db. Additional file ml2db_code.tar.gz contains the Java source code to run the multi-label machine learning experiments and save the results to database. The code’s Javadoc is included. [file 1471-2105-15-150-S2.zip › additional file 2/ml2db/ml2db/doc/mulan/classifier/package-use.html]

Uses of Package mulan.classifier


JavaScript is disabled on your browser.


- Overview
- Package
- Class
- Use
- Tree
- Deprecated
- Index
- Help

- Prev
- Next

- Frames
- No Frames

- All Classes

# Uses of Package mulan.classifier

- Packages that use mulan.classifier

  | Package | Description |
  | --- | --- |
  |  |  |
  | --- | --- |
  | mulan.classifier |  |
  | mulan.classifier.lazy |  |
  | mulan.classifier.meta |  |
  | mulan.classifier.meta.thresholding |  |
  | mulan.classifier.neural |  |
  | mulan.classifier.transformation |  |
  | mulan.data |  |
  | mulan.evaluation |  |
  | mulan.evaluation.loss |  |
  | mulan.evaluation.measure |  |
- Classes in mulan.classifier used by mulan.classifier

  Class and Description

  InvalidDataException

  Exception thrown by the `MultiLabelLearner` when presented with invalid data or
  data which can not be processed by the specific learner type.

  LearnerException

  Represents a root base class for exceptions thrown by learners.

  ModelInitializationException

  Exception thrown by the `MultiLabelLearner` when learner is queried for prediction on data
  before model is internally built by learning from training data.

  MultiLabelLearner

  Common root interface for all multi-label learner types.

  MultiLabelOutput

  Class representing the output of a `MultiLabelLearner`.
- Classes in mulan.classifier used by mulan.classifier.lazy

  Class and Description

  MultiLabelLearner

  Common root interface for all multi-label learner types.

  MultiLabelLearnerBase

  Common root base class for all multi-label learner types.
- Classes in mulan.classifier used by mulan.classifier.meta

  Class and Description

  MultiLabelLearner

  Common root interface for all multi-label learner types.

  MultiLabelLearnerBase

  Common root base class for all multi-label learner types.

  MultiLabelOutput

  Class representing the output of a `MultiLabelLearner`.
- Classes in mulan.classifier used by mulan.classifier.meta.thresholding

  Class and Description

  MultiLabelLearner

  Common root interface for all multi-label learner types.

  MultiLabelLearnerBase

  Common root base class for all multi-label learner types.

  MultiLabelOutput

  Class representing the output of a `MultiLabelLearner`.
- Classes in mulan.classifier used by mulan.classifier.neural

  Class and Description

  InvalidDataException

  Exception thrown by the `MultiLabelLearner` when presented with invalid data or
  data which can not be processed by the specific learner type.

  MultiLabelLearner

  Common root interface for all multi-label learner types.

  MultiLabelLearnerBase

  Common root base class for all multi-label learner types.

  MultiLabelOutput

  Class representing the output of a `MultiLabelLearner`.
- Classes in mulan.classifier used by mulan.classifier.transformation

  Class and Description

  MultiLabelLearner

  Common root interface for all multi-label learner types.

  MultiLabelLearnerBase

  Common root base class for all multi-label learner types.

  MultiLabelOutput

  Class representing the output of a `MultiLabelLearner`.
- Classes in mulan.classifier used by mulan.data

  Class and Description

  MultiLabelLearner

  Common root interface for all multi-label learner types.
- Classes in mulan.classifier used by mulan.evaluation

  Class and Description

  MultiLabelLearner

  Common root interface for all multi-label learner types.
- Classes in mulan.classifier used by mulan.evaluation.loss

  Class and Description

  MultiLabelOutput

  Class representing the output of a `MultiLabelLearner`.
- Classes in mulan.classifier used by mulan.evaluation.measure

  Class and Description

  MultiLabelOutput

  Class representing the output of a `MultiLabelLearner`.

- Overview
- Package
- Class
- Use
- Tree
- Deprecated
- Index
- Help

- Prev
- Next

- Frames
- No Frames

- All Classes
